# Pre-steady-state Kinetic Analysis of Amino Acid Transporter SLC6A14 Reveals Rapid Turnover Rate and Substrate Translocation

**DOI:** 10.3389/fphys.2021.777050

**Published:** 2021-11-16

**Authors:** Yueyue Shi, Jiali Wang, Elias Ndaru, Christof Grewer

**Affiliations:** Department of Chemistry, Binghamton University, Binghamton, NY, United States

**Keywords:** membrane transporter, SLC6A14, ATB^0,+^, electrophysiology, rapid kinetics, laser-photolysis, molecular physiology

## Abstract

SLC6A14 (solute carrier family 6 member 14) is an amino acid transporter, driven by Na^+^ and Cl^−^ co-transport, whose structure, function, and molecular and kinetic mechanism have not been well characterized. Its broad substrate selectivity, including neutral and cationic amino acids, differentiates it from other SLC6 family members, and its proposed involvement in nutrient transport in several cancers suggest that it could become an important drug target. In the present study, we investigated SLC6A14 function and its kinetic mechanism after expression in human embryonic kidney (HEK293) cells, including substrate specificity and voltage dependence under various ionic conditions. We applied rapid solution exchange, voltage jumps, and laser photolysis of caged alanine, allowing sub-millisecond temporal resolution, to study SLC6A14 steady state and pre-steady state kinetics. The results highlight the broad substrate specificity and suggest that extracellular chloride enhances substrate transport but is not required for transport. As in other SLC6 family members, Na^+^ binding to the substrate-free transporter (or conformational changes associated with it) is electrogenic and is likely rate limiting for transporter turnover. Transient current decaying with a time constant of <1ms is also observed after rapid amino acid application, both in forward transport and homoexchange modes, indicating a slightly electrogenic, but fast and not rate-limiting substrate translocation step. Our results, which are consistent with kinetic modeling, suggest rapid transporter turnover rate and substrate translocation with faster kinetics compared with other SLC6 family members. Together, these results provided novel information on the SLC6A14 transport cycle and mechanism, expanding our understanding of SLC6A14 function.

## Introduction

Plasma membrane amino acid transporters move amino acids across the lipophilic membrane bilayer into the cell (Zafra and Gimenez, 1986; Reyes et al., 2009; Wang et al., 2018), providing nutrients for many essential biological processes, such as protein and nucleotide synthesis, mammalian target of rapamycin (mTOR) signaling, and cell metabolism. Amino acid transporters belong to several families of solute carrier (SLC) proteins and are differentiated by sequence similarity, substrate specificity, ion dependency, and transporter mechanism (Broer and Gether, 2012; Schaller and Lauschke, 2019), which contribute to various aspects of transporter function and regulation. Amino acid transporter SLC6A14 belongs to the Solute Carrier 6 (SLC6) family, which consists of 20 membrane transporters in the human genome. It is also known as ATB^0,+^ derived from transport selectivity for neutral amino acids, denoted by “0” and cationic amino acids denoted by “+.” SLC6A14 is a unidirectional transporter, in which amino acid transport is coupled to Na^+^ and Cl^−^ co-transport, with broad substrate selectivity (Sloan and Mager, 1999; Ugawa et al., 2001; Le Guellec et al., 2021), including all neutral and cationic amino acids. It was also identified as a β-alanine carrier (Bhutia et al., 2015). The SLC6 family transporters are subdivided into four branches, based on sequence similarity, which are monoamine transporters, GABA transporters and amino acid transporters (I) and (II) (Page, 1996; Notredame et al., 2000; Broer and Gether, 2012). The amino acid SLC6 transporters from subfamily (I) transport glycine (GlyT1/SLC6A9, GlyT2/SLC6A5), proline (PROT/SLC6A7) and neutral/cationic amino acids (ATB^0,+^/SLC6A14). Amino acid transporter subfamily (II) comprised nutrient amino acid transporters SLC6A15-20 (Pramod et al., 2013; Rudnick et al., 2014; Coothankandaswamy et al., 2016).

The crystal structure of a bacterial homolog of SLC6 bacterial leucine transporter, LeuT (Yamashita et al., 2005), and a drosophila dopamine transporter (Penmatsa et al., 2013), as well as biochemical research (Kristensen et al., 2011), provided a foundation of structural and functional properties of SLC6 family members. While no crystal structure is known for SLC6A14, a homology model based on *Drosophila melanogaster* sodium-dependent DAT was published (Palazzolo et al., 2019), and another model is available from the AlphaFold server (Jumper et al., 2021). In contrast to the structure, the SLC6A14 functional properties have been investigated in more detail. SLC6A14 amino acid transport is dependent on Na^+^ and Cl^−^, with a proposed stoichiometry of 2:1:1 (Na^+^: amino acid: Cl^−^; Nakanishi et al., 2001; Umapathy et al., 2004; Karunakaran et al., 2011). Therefore, amino acid transport is electrogenic and associated with transport current, which was measured in *Xenopus* oocytes.

Amino acid transporter SLC6A14 has been cloned from human and rat tissues (Sloan and Mager, 1999; Nakanishi et al., 2001; Ugawa et al., 2001; Umapathy et al., 2004). SLC6A14 is highly expressed in lung, intestine, and other tissues such as pituitary, colon, and mammary gland. SLC6A14 is proposed to be upregulated in several cancers with the purpose of delivering amino acid nutrients into the cells, including colorectal cancer (Gupta et al., 2005), cervical cancer (Gupta et al., 2006), estrogen receptor positive breast cancer (Karunakaran et al., 2011; Schaller and Lauschke, 2019), and pancreatic cancer (Penheiter et al., 2015; Coothankandaswamy et al., 2016). The inhibition of SLC6A14 decreases the proliferation of tumor cells either with shRNA-mediated gene silencing or using α-methyl-L-tryptophan as an inhibitor *in vitro* and *in vivo* (Karunakaran et al., 2008; Babu et al., 2015). Therefore, SLC6A14 has great potential for further investigation as a drug target for cancer treatment.

Currently, the details of the transport mechanism of SLC6A14, as well as kinetic parameters, such as turnover rate, are unknown. Here, we expressed SLC6A14 in human embryonic kidney (HEK) cells to investigate substrate specificity under different ionic conditions, as well as the voltage dependencies of individual steps in the transport cycle. We further applied laser pulse photolysis with a caged amino acid, to investigate its functional and kinetic properties in steady state and pre-steady state. Consistent with other SLC6 family members, binding of extracellular Na^+^ is a major contributor to transporter voltage dependence and limiter of the turnover rate (Hilgemann and Lu, 1999; Mennerick et al., 1999; Sloan and Mager, 1999; Grewer et al., 2000b, 2012; Watzke et al., 2001; Zhang et al., 2007; Wang et al., 2021). The anion effect, however, is more subtle, with Cl^−^ not being required for, but facilitating transport kinetically. Turnover rate was found to be one of the fastest in the SLC6 family.

## Materials and Methods

### Cell Culture and Transfection

Human embryonic kidney 293 (HEK293, American Type Culture Collection CRL-1573) cells were cultured in DMEM prepared with 45g/L fetal bovine serum (FBS), 4.5g/L of penicillin, L-glutamine sodium pyruvate, and non-essential amino acid mix (Gibco) in the fully humidified incubator with constant temperature of 37°C and 5% carbon dioxide. Cell cultures were transiently transfected with wild-type SLC6A14 and YFP cDNAs (Genecopoeia, TakaraBio) using jet-PRIME transfection reagent followed by the protocol supplied by POLYPLUS-Transfection. Cells were incubated for 20–30h after transfection and ready for electrophysiology technique.

### Electrophysiological Techniques

The whole-cell recording configuration was used to perform electrophysiological experiments. The external buffer solutions used to measure the transport current were prepared in 140mM NaCl, NaMes, NaI or NaGluconate, 2mM MgCl_2_ or Mg(gluconate)_2_, 2mM CaCl2/ CaMes/Ca(gluconate)_2_, and 10mM HEPES in pH 7.40. When changing the anion at 140mM Na^+^, their respective salts were used, i.e., NaCl for Cl^−^, and NaI for I^−^, or a 140mM NaOH solution was titrated with gluconic acid or methanesulfonic acid. The internal buffer KMes/ KSCN/KGluconate were prepared from 130mM KMes or KSCN or KGluconate, 2mM MgCl_2_ or Mg(gluconate)_2_, 10mM EGTA, and 10mM HEPEs, pH 7.40, as published earlier (Grewer et al., 2001). To analyze anion effects in SLC6A14 transporters, currents at different extracellular or intracellular anion compositions, including various external or internal buffers, were recorded, respectively. Whole-cell recordings method were conducted in similar fashion as in previous publications (Wang et al., 2019; Grewer et al., 2000b; Wang et al., 2020). The glass patch pipettes back-filled with internal buffer (open pipette resistance of 3–7 MΩ) were used to establish a seal with the cell membrane (resistance in the 500 MΩ to GΩ range). After establishing the whole cell recording mode using voltage pulses and/or suction, currents associated with the SLC6A14 amino acid transporter were recorded with an Adams & List EPC7 patch-clamp amplifier and digitized using a Molecular Devices Digidata A/D converter connected to a computer.

### Voltage Jump Experiments

Voltage jumps (−100 to +60mV) were performed on SLC6A14-expressing cells to perturb the electrogenic substrate (alanine) transport steady state, or the equilibria associated with partial reactions, such as Na^+^ binding. To determine Na^+^-dependent SLC6A14 currents, control currents were recorded in the presence of various Na^+^ concentrations and subtracted from the substrate-induced currents. 5, 70,140mM Na^+^ solutions were made from 140mM NaCl and NMGCl buffers and the cells were immersed in NMGMes bath. To determine anion effects on SLC6A14, control currents were recorded in the presence of external buffers containing various anion concentrations and subtracted from the substrate-induced currents. In addition, α-Methyl-Tryptophan was used as an inhibitor for subtracting unspecific currents. Capacitive transient compensation and series resistance compensation of up to 80% were achieved with EPC7 amplifier. Clampfit (Molecular Devices) was used to do subtraction of non-specific transient currents.

### Rapid Solution Exchange and Laser Photolysis of Caged-Compounds

Rapid solution exchange (time resolution 100–200ms) was performed by means of a quartz tube (opening diameter 350μM) positioned at 0.5mM to the cell. The solution flowed to the opening of the tube with the linear flow rate of 5–10cm/s. Laser-pulse photolysis experiment were performed as described in detail previously (Niu et al., 1996; Grewer et al., 2001; Watzke et al., 2001; Tao et al., 2006; Wang et al., 2021). Photolysis of caged compounds was activated with a light flash (355nm, 8ns, frequency-tripled Minilite II, Continuum), which was delivered from a quartz fiber (diameter 365μM), and placed in front of the cell at a distance of 300μM. With laser light intensities of 500–840mJ/cm^2^, saturated free alanine or serine could be released, as tested in comparison with steady-state currents elicited by a known concentration of alanine, as described previously (Grewer et al., 2001). Data were recorded using pClamp6 software (Axon Instruments), digitized with a sampling rate of 1kHz (solution exchange) or 25kHz (laser photolysis and voltage jump) and low-pass filtered at 3–10kHz.

### Piezo-Based Solution Switching

Fast solution exchanges were applied using the setup SF-77B (Warner Instruments, LLC, MA, United States), allowing a time resolution in the 10–20ms range (details can be found in the manufacturer’s manual). The experiments were tested under forward transport conditions, extracellular with 140mM NaCl, intracellular with 130mM KMes. Five mM of alanine was rapidly applied through a theta capillary glass tubing (TG200-4, OD=2.00mM, ID=1.40mM, Warner Instruments, LLC, MA, United States), currents were recorded using pClamp software and analyzed in Origin software.

### Data Analysis

All data are shown as mean±SD, collected from recordings of 8–10 cells. For statistical analysis, paired two-tailed *t* tests were used in Microcal Origin software. To determine substrate K_m_ values, non-linear curve fitting was used with a Michaelis-Menten-like equation, y=I_max_^*^[substrate]/(K_m_+[substrate]) built in the Origin software least-squares-fitting package.

Nonlinear regression fits of laser-pulse photolysis experimental results were performed in Clampfit software (Axon Instruments) using the following equations. The pre-steady-state transport currents (in the absence of SCN^−^) were fitted with a sum of two exponential functions and a steady-state current component: I=I1·exp(−t/τrise)+I2·exp(−t/τdecay1)+ISS. Here, *I* is the current amplitude, *τ* the time constant, and *t* the time.

### Synthesis of Caged Alanine

**Figure 1:**
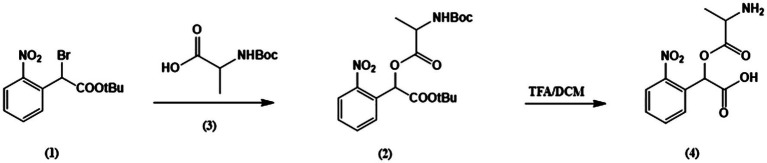
Synthesis scheme of caged alanine.

The caged alanine was synthesized according to the following procedures, as illustrated in the reaction scheme 1 (Figure 1).

Tert-butyl-2-bromo-2-(2-nitrophenyl)acetate (1). General synthesis of this intermediate was performed as described previously (Grewer et al., 2001).

2-(tert-butoxy)-1-(2-nitrophenyl)-2-oxoethyl (tert-butoxycar bonyl) alaninate (2). A mixture of N, N-diisopropylethylamine (204mg, 1.58mmol, 5 equi.), tert-butyl-2-bromo-2-(2-nitrophenyl)acetate (1) (100mg, 0.316mmol, 1 equi.) and tert-butoxycarbonyl alanine (65.8mg, 0.348mmol, 1,1 equi. (3) were weighed into an oven-dried round bottom flask. Acetone was added through a syringe into the flask purged with N_2_ gas and the solution was refluxed at 60°C. The reaction mixture was stirred overnight and monitored by thin-layer chromatography (TLC) to reach completion. Subsequently, all contents were washed twice with sodium bicarbonate, twice with water. The organic layer was dried over sodium sulfate and filtered. The filtrate was concentrated *in vacuo* and product was purified using silica gel chromatography (0–25% ethyl acetate in hexane) to obtain a pure oil. 1H NMR (400MHz, CDCl3) δ 8.03 (dd, J=8.4, 2.0Hz, 1H), 7.75–7.61 (m, 2H), 7.54 (ddd, J=9.9, 6.8, 3.7Hz, 1H), 6.79 (d, J=15.5Hz, 1H), 5.09 (s, 1H), 4.42 (dd, J=47.5, 8.5Hz, 1H), 1.58–1.49 (m, 2H), 1.45 (s, 1H), 1.40 (dd, J=12.4, 7.3Hz, 18H).

2-(alanyloxy)-2-(nitrophenyl) acetic acid (4). The protected compound (2) (72mg, 0.17mmol, 1.0 equi.) and Dithiothreitol (DTT; 52mg, 0.34mmol, 2 equi.) were weighed into an oven-dried round bottom flask. Trifluoroacetic acid (0.42ml, 5.4mmol, 32 equi.) was added dropwise under N_2_ gas using syringe and cooled to 0°C to room temperature. The reaction mixture was stirred for 46h which monitored by TLC (20–40% MeOH in DCM). TFA was removed under high vacuum and the mixture was concentrated *in vacuo*. The desired product was purified as a white solid through trituration in chilled diethyl ether and confirmed by TLC (20–40% MeOH in DCM). The stock solution in external buffer was prepared for further laser experiments.

## Results

### Amino Acid Substrate Selectivity in SLC6A14

**Figure 2:**
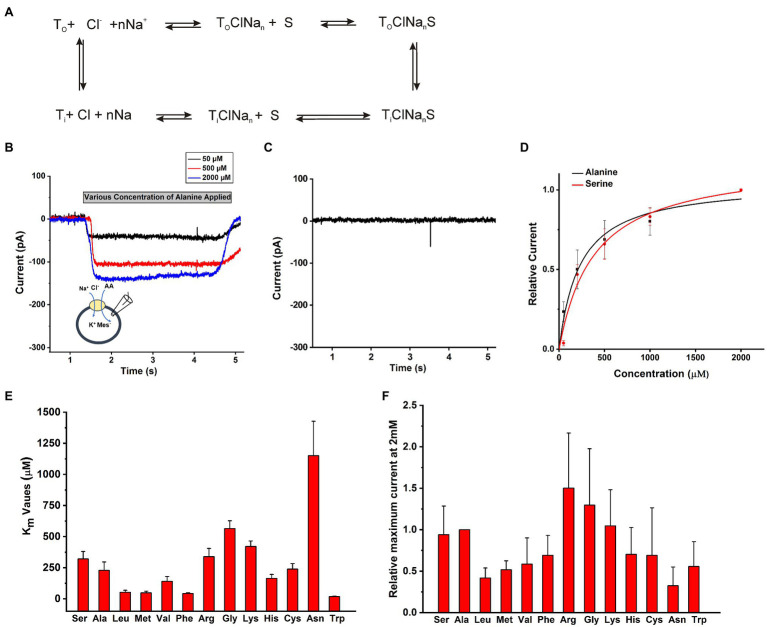
Amino acid substrate selectivity. **(A)** A conceptual transport cycle of SLC6A14. **(B)** Typical electrophysiological recordings of concentration-dependent alanine-induced currents under forward transport conditions (140mM NaCl as external buffer and 130mM KMes as internal buffer). The time of application of substrate is indicated by the grey bar. Due to slight variations in the rate of flow in each tube for solution exchange, and the response time of the pinch valves, there is some variation in the onset of the current signal for each concentration. **(C)** An example trace of 200μM alanine on HEK cells not expressing SLC6A14. **(D)** Alanine and serine dose response curves. The black (alanine) and red (serine) lines represent the best non-linear least squares fit to a Michaelis–Menten-like equation with apparent Km value of 210±40μM for alanine and 320±60μM for serine. **(E)** Apparent Km values for various amino acids. **(F)** Inward transport currents (realive to alanine) at 2mM concentration of amino acid. The membrane potential was 0mV in all experiments.

The objective of this work was to determine the functional properties of SLC6A14 by electrophysiology in a cell system that allows control of the ionic composition of solutions on both sides of the membrane. A conceptual transport cycle for SLC6A14 is shown in Figure 2A. To obtain the details of kinetic properties, we selected HEK293 cells to transiently over-express SLC6A14 cDNA and then voltage clamped the cells in the whole-cell recording mode. In contrast to previously-used *Xenopus* oocyte system, the intracellular solution can be manipulated through the ionic composition of the patch-clamp pipette in whole cell recordings of HEK293 cells. To compare the transport specificity of SLC6A14 with respect to different amino acids in HEK cells with respect to previously published data from the *Xenopus* oocyte system (Sloan and Mager, 1999; Nakanishi et al., 2001; Ugawa et al., 2001; Hatanaka et al., 2004; Karunakaran et al., 2008), cells were voltage clamped at 0mV and solutions were applied to cells using a fast solution exchange setup. As expected from the electrogenicity of SLC6A14, extracellular application of the prototypical amino acid alanine resulted in inward currents (typical current recordings are shown in Figure 2B). Non-transfected cells did not show any currents induced by alanine (Figure 2C). Inward currents increased with increasing alanine concentrations (Figures 2B,D). These currents are expected to be caused by the stoichiometric, net ionic charge movement of Na^+^ into the cell, while being co-transported with the neutral amino acid molecule and Cl^−^. Typical dose response curves for L-alanine and L-serine as substrates are shown in Figure 2D with 140mM NaCl external and 130mM KMes internal buffers. After fitting to a Michaelis–Menten equation, the K_m_ for substrate Ala and Ser were determined to be 210±40μM and 320±60μM, respectively. Other amino acids’ apparent affinity was tested in the same way, and the results are summarized in Figure 2E. Trp, Leu, Met, and Phe showed the highest apparent affinities (Supplementary Table S1) while Ala, Ser, Val, and Cys had intermediate level K_m_ values. In contrast, the basic amino acids, Arg and Lys, together with Gly and Asn had the lowest apparent affinities in the >350μM range (Figure 2E, Supplementary Table S1). Amino acids with negatively-charged side chain did not elicit currents. In addition, aspartate at a concentration up to 500μM, was unable to block alanine-induced transport current (200μM), indicating that acidic amino acids are not recognized by the SLC6A14 binding site. Overall, aromatic amino acids with hydrophobic side chain have higher apparent affinities. Absolute currents magnitudes were analyzed at 2mM substrate concentration and shown in Figure 2F. This concentration was chosen because it is close to saturating for most but the lowest affinity substrates. From comparison of these currents’ magnitudes, the lower affinity amino acids Arg and Gly induced the largest currents. These results are generally consistent with those from the literature (Sloan and Mager, 1999; Ugawa et al., 2001; Karunakaran et al., 2008; Fairweather et al., 2021), although the exact apparent K_m_ values are slightly different, as is often the case when comparing results from mammalian cell lines to those from *Xenopus* oocytes.

### Extracellular Chloride Is Not Required for, but Enhances Substrate Transport

**Figure 3:**
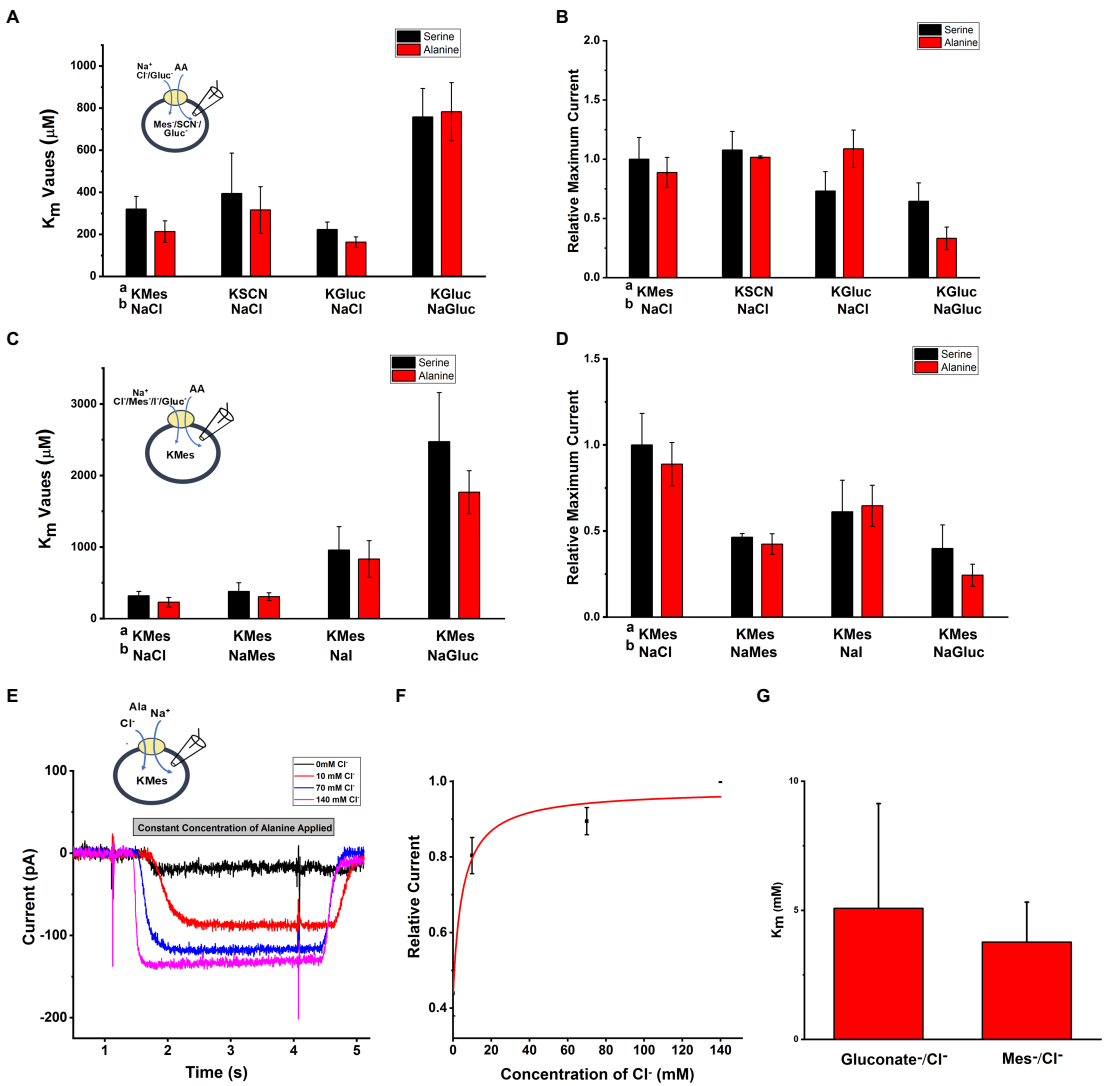
Anion effect on alanine and serine transport currents. A Intracellular buffer; b extracellular buffer. **(A)** Apparent affinities for alanine or serine at different anionic conditions at the intracellular side. Internal anion concentration was 130mM. The extracellular solution contained 140mM NaCl, except for one experiment external NaGluc. **(B)** Current induced by alanine and serine (2mM concentration) under the same conditions as in **(A)**. **(C)** Apparent affinities for alanine or serine when varying the anion at the extracellular side. **(D)** Inward current induced by alanine and serine (2mM concentration) under the same conditions as in **(C)**. **(E)** Typical electrophysiological recordings of alanine-induced currents at increasing concentrations of cloride (NaGluc replacing NaCl). The alanine concentration was kept the same at 2mM. **(F)** Dose response curve for chloride with K_m_ of 5.1±2.4mM. **(G)** Apparent K_m_ for Cl^−^ with replacement by either Mes^−^ or Gluc^−^. The voltage was 0mV in all experiments.

To test the effect of anions on SLC6A14 transporter properties, we used whole-cell recording method with external and internal buffers containing various types of anions. Upon applying the amino acid from the extracellular side, inward currents were activated with or without extracellular or intracellular Cl^−^ (Figures 3A, B). When extracellular Cl^−^ was replaced with large, organic anions that are typically not known to interact with Cl^−^ binding sites (Sloan and Mager, 1999; Nakanishi et al., 2001; Hatanaka et al., 2004), such as Mes^−^ and Gluconate^−^ (Gluc^−^), inward current was reduced, but not abolished (Figure 3D). However, the apparent affinity for the amino acid substrate was reduced, in particular in the presence of extracellular Gluconate^−^ (Figure 3C). Then, we analyzed the [Cl^−^] dependence of transport current at a saturating concentration of alanine (Figures 3E–G). This is done by monitoring the inward current of alanine in presence of increasing concentration of Cl^−^. When we selected different anions to replace Cl^−^, Mes^−^, and Gluc^−^ showed different effects. The Michaelis constant (K_m_) for Cl^−^ in presence of NaGluc^−^ was 5.1±2.4mM and for NaMes was 3.8±1.6mM. The result from Figure 3E show that even with 0mM concentration of external Cl^−^, inward currents were still generated by alanine application in the presence Mes^−^ or Gluconate^−^.

### SLC6A14 Amino Acid Transport Is Na^+^ Dependent

**Figure 4:**
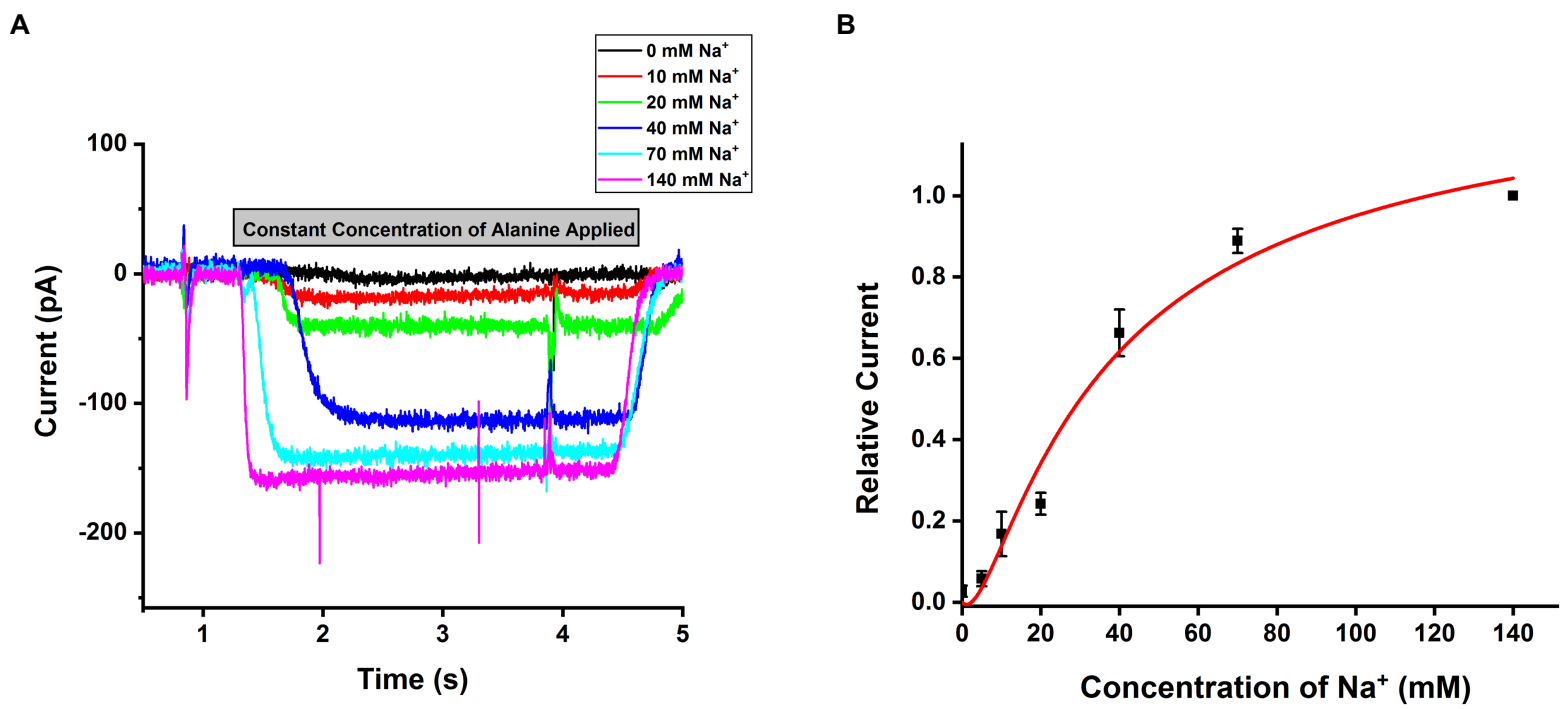
Na^+^ dependence of alanine mediated SLC6A14 transport currents. **(A)** Typical alanine-induced transport currents (2mM alanine) at varying Na^+^ concentrations (replaced by NMG+). The internal buffer contained 130mM KMes. **(B)** Na^+^ dose response curve under the same conditions as in **(A)**. The results were fitted using the Hill equation (solid line). The fit parameters were Km=32.2±4.3mM, and *n*=1.96. The voltage was 0mV in all experiments.

SLC6A14 was reported as a sodium and chloride co-transporter with a Na^+^: Cl^−^ stoichiometry of 2:1 (Nakanishi et al., 2001; Umapathy et al., 2004; Karunakaran et al., 2011). To test this hypothesis, we analyzed the [Na^+^] dependence of transport currents at a saturating concentration of alanine. An example of representative currents is shown in Figure 4A, with currents showing strong [Na^+^] dependence with constant alanine and being virtually eliminated in the absence of sodium (replaced with NMG^+^). The Na^+^ dependence could be fitted using a Hill equation with a Hill coefficient of 1.96±0.3, as expected for the 2:1 Na^+^ to substrate stoichiometry (Figure 4B). The apparent affinity for sodium was K_m_=32.2±4.3mM, shown in Figure 4B. Together, these results confirm SLC6A14 to be a Na^+^-dependent amino acid symporter.

### Voltage Dependence of SLC6A14 Currents

**Figure 5:**
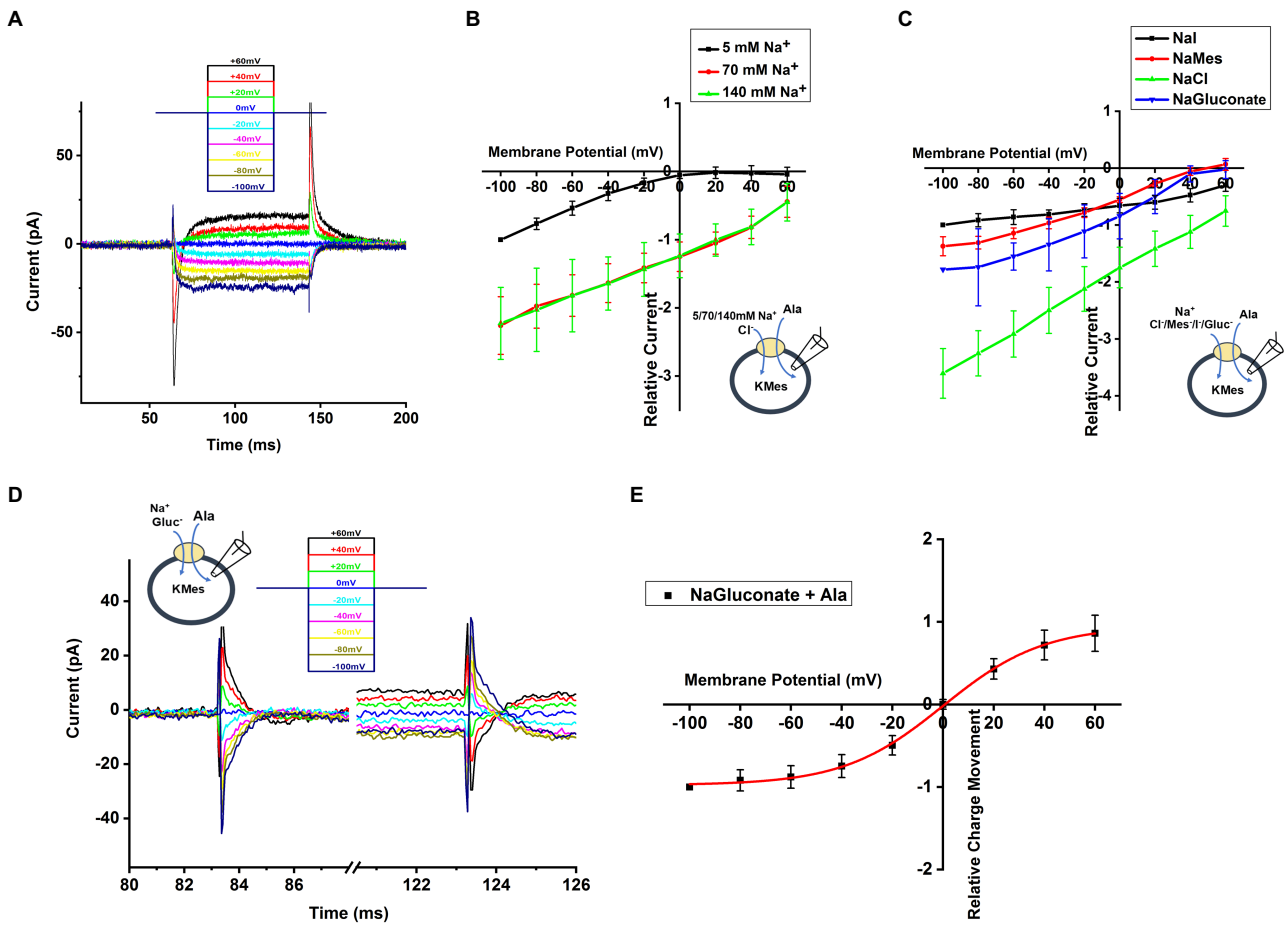
Voltage Dependence of SLC6A14 currents. **(A)** Typical transport current recordings at different membrane potentials, ranging from −100mV to 60mV in the presence of 130mM of intracellular KMes and 10mM alanine in the external buffer (140mM NaCl). The voltage jump protocol is illustrated in the inset (top). **(B)** Voltage dependence of relative transport currents as a functiom of the external [Na^+^]. The concentration of 2mM alanine was kept constant. Specific currents were calculated after subtraction of currents in the presence of inhibitor α-Methyl-Tryptophan, to eliminate background currents. All currents were normalized to the current induced at 5mM Na^+^ at −100mV. **(C)** Currents similar as in **(B)**, but with different anions in the extracellular solution. **(D)** Voltage dependence of the transient current component. **(E)** Integration of the transient current results in the transient charge movement, relative to the charge movement at −100mV.

To determine the voltage dependence of alanine transport, we measured currents after jumping the voltage from 0mV to a range from −100mV to +60mV (voltage protocol shown in Figure 5A, top). Currents in the absence of alanine were then subtracted from currents in the presence of alanine to generate the amino acid-specific signal. As shown in Figure 5A, a voltage-jump to negative potentials induced a rapid transient current, followed by relaxation to a steady-state inward transport current. This steady-state current relaxed to baseline upon jumping the voltage back to 0mV. The voltage dependence of the steady-state transport current was analyzed by subtracting current records during a series of voltage jumps in the presence of inhibitor α-Methyl-Tryptophan from corresponding currents in the presence of alanine only. As shown in Figure 5B, the relative transport current increased almost linearly with more negative membrane potential at 140 and 70mM extracellular [Na^+^]. In contrast, transport current was almost absent at positive potentials in the presence of low Na^+^ concentration (5mm), but increased exponentially at negative membrane potentials, suggesting a voltage dependence of the K_m_ for Na^+^ (higher apparent affinity at negative voltage and lower affinity at positive voltage), as expected if Na^+^ binding is electrogenic, as is the case in other SLC6 family members (Jiang et al., 2005; Erdem et al., 2019).

Next, we further analyzed voltage dependence of transport current in the presence of different extracellular anions, as shown in Figure 5C. These results confirm the previous observations at 0mV, namely that Cl^−^ is not required for amino acid transport process, but that it promotes transport currents at all membrane potentials. Interestingly, the voltage dependence in the presence of iodide is much weaker than with the other anions (Figure 5C). These results could indicate that a voltage-independent step becomes rate limiting in the presence of I^−^.

To determine the relative charge movement of the transient current induced by the voltage jumps, we integrated the area of inward-directed transient current over time, which is shown in Figure 5D. No matter whether hyperpolarizing and depolarizing voltage pulses were applied, the charge movement induced by initiating the voltage jump (on charge) was equivalent to the charge movement after returning to the original potential (off charge). When plotting charge-voltage relationship in Figure 5E, we observed that the relative charge movement saturated at negative potential (< −80mV) and increased steeply in the range of −40mV to +40mV. The Q-V curve was fitted using a Boltzmann-like function with a midpoint potential of 0.11mV and slope factor of 50.6V^-1^.

**Figure 6:**
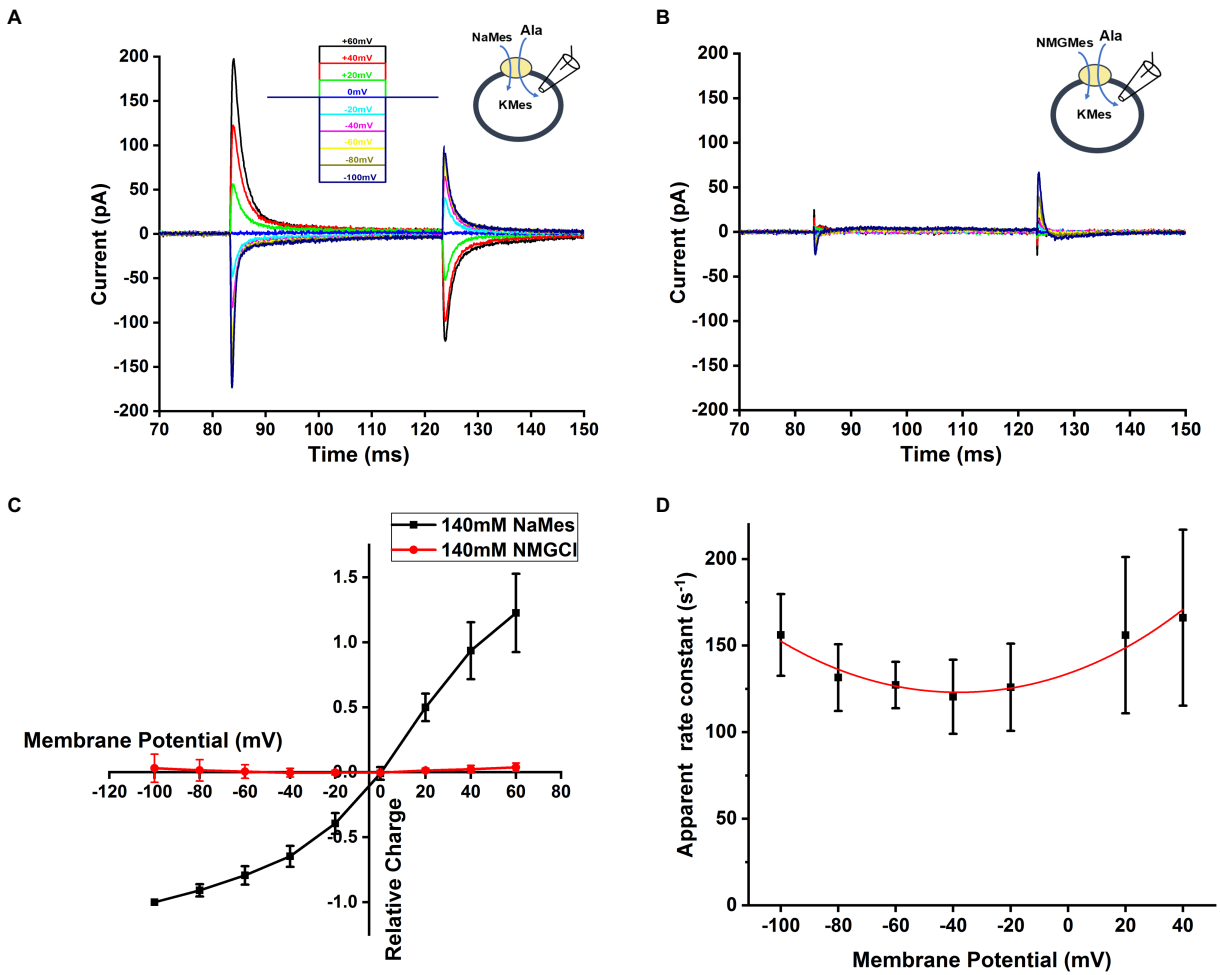
Na^+^-dependent transient currents in the absence of substrate. **(A)** Typical transient currents in response to voltage jumps from 0mV to different membrane potential, ranging from −100mV to +60mV, in the presence of extracellular 140mM NaMes and intracellular 130mM KMes. The concentration of alanine was 1mM. The voltage jump protocol is illustrated in the inset (top). **(B)** Similar experiment as in (A), but Na^+^ was replaced by NMG+. **(C)** The voltage dependence of the transient charge movement was analyzed by subtracting currents in response to a series of voltage jumps in the presence of α-Methyl-Tryptophan from corresponding currents in the absence of α-Methyl-Tryptophan, to eliminate any un-specific background currents. The charge-voltage relationship at 140mM NaMes is shown in black, and at 140mM NMGCl in red. Based on Boltzmann equation $Q\left(V_{m}\right)=Q_{\min}+\frac{Q_{\max}}{1+e^{\frac{Z_{Q}\left(V_{Q}-V_{m}\right)•F}{RT}}}$ , where *Q* represents the charged moved, *z* is valence of charge moved, V is the membrane potential. The valence obtained from the fit was 0.9. **(D)** Voltage dependence of the relaxation rate constant of voltage jump-induced transient current decay fitted using equation is $k_{obs}=k_{for}^{0}e^{\frac{-zFV}{2RT}}+k_{rev}^{0}e^{\frac{zFV}{2RT}}\;$ with $k_{for}^{0}\;$ as forward intrinsic binding rate, $k_{rev}^{0}$ as reversed intrinsic dissociation rate and *z* as valence of the charge movement, *z*=0.56.

In other SCL6 members, pre-steady state charge movement was observed in the absence of substrate and is thought to be related to Na^+^ binding (Mager et al., 1996; Hilgemann and Lu, 1999; Erdem et al., 2019). In order to isolate transient charge movement in the absence of substrate, we applied the SLC6A14 blocker, α-Methyl-Tryptophan, to analyze the current induced by voltage jump without back-ground current. In this experiment, the voltage dependence of the transport current was analyzed by subtracting current records during a series of voltage pulses in the presence of α-Methyl-Tryptophan from corresponding currents in the absence of α-Methyl-Tryptophan to strongly inhibit transporter to eliminate any back-ground current effect. An example trace of transient current induced by voltage jumps at 140mM NaMes condition is shown in Figure 6A. The transient current is Na^+^ dependent, because it is eliminated in the presence of 140mM NMGCl as shown in Figure 6B. The current decayed with a time constant of 7.1±1.0ms at 0mV, and the decay is accelerated at negative membrane potentials. The charge-voltage relationship, obtained by integrating the current, is plotted using a Boltzmann-like function with a midpoint potential of 13.5mV and slope factor of 35.2V^-1^ in Figure 6C, pointing to a valence of 0.9 for the underlying electrogenic process. The charge movement starts to saturate at negative −100mV membrane potentials. Overall, these results are compatible with voltage-dependent Na^+^ binding step(s), or conformational changes associated with them, for example, a potential redistribution of the inward-outward-facing equilibrium of the empty transporter. Figure 6D shows the dependence of the rate constant for the transient current decay as a function of the voltage. As expected for a reversible, electrogenic reaction, the rate constant increases at both positive and negative voltages, and displays a minimum at −40mV.

### Pre-steady-state Currents in Response to Rapid Application of Alanine Through Photolysis of Caged Alanine

Previously, SLC6A14 pre-steady-state currents could not be measured in response to the application of alanine, due to limitations in the rate of solution exchange when using *Xenopus* oocytes (Sloan and Mager, 1999; Nakanishi et al., 2001; Ugawa et al., 2001; Umapathy et al., 2004; Karunakaran et al., 2008, 2011), due to their large diameter of approximately 1mM. To circumvent this problem, we synthesized caged alanine (see synthesis scheme, Figure 1) as a tool to rapidly liberate free alanine through flash photolysis, allowing the detection of early steps in the amino acid transport cycle. We first examined the effect of the caged compound on SLC6A14 transporter steady-state currents, showing no change of the magnitude of alanine-induced inward current with or without caged alanine, suggesting that the compound is biologically inert with respect to the SLC6A14 transporter. We observed small inward current before light activation of caged alanine, which contributed to small inward background currents caused by a background of free alanine in the caged compound, either from the purification protocol, or through some spontaneous hydrolysis of caged alanine (<50μM).

**Figure 7:**
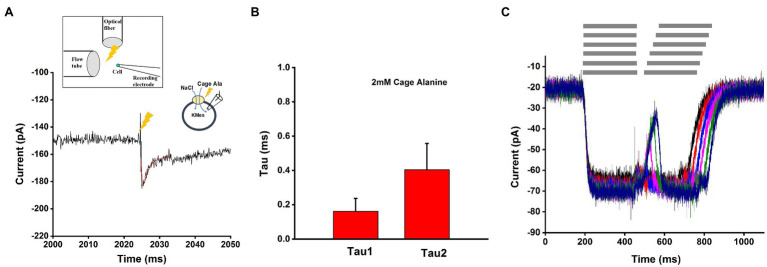
Pre-steady state currents in response to alanine concentration jumps. **(A)** Application of the substrates with rapid solution exchange by photolysis of 2mM caged alanine under forward transport conditions. The time of the laser flash is indicated by the arrow. Transient current was fitted with a sum of two-exponential functions, as shown by the red line. The time constants from fitted function were 0.17ms (rising phase) and 0.43ms (decay phase). The external buffer contained 140mM NaCl and the internal buffer contained 130mM KMes. **(B)** Average of the time constants obtained from the fit to responses from 15 cells of 0.16±0.07ms (rising phase) and 0.40±0.15ms (decaying phase). **(C)** Paired-pulse solution exchange experiments with a piezo-based solution switching system (10–20ms time resolution) were performed under the same ionic conditions as **(A)** and **(B)**. Example of currents was recorded with 20ms interval time. The gray bar on the top of **(C)** indicted the rapid solution exchange to substrate contained solution.

After photolysis with a brief laser flash, a pre-steady-state inward current was observed, which rapidly decayed to the steady state within less than 10ms (Figure 7A) under forward transport conditions (intracellular KMes). Using a sum of two exponential components and a steady state for fitting, time constants of 0.16ms (rising phase) and 0.40ms (decaying phase) were observed at 2mM caged alanine, as shown in Figure 7B. The liberated alanine concentration was estimated to about 300μM, not a saturating concentration, but one that is > K_m_.

We also used a piezo-driven rapid solution exchange device to test the recovery of the current using paired pulses, as well as the rate constant of current decay upon alanine removal, as a potential determinant of the turnover rate. As shown in Figure 7C, despite its 10–20ms time resolution, the method was unable to resolve the transient current and the recovery rate in time, most likely due to the very rapid transporter kinetics. However, from the deactivation of the current after alanine removal, a decay rate of 19±5ms was determined. This is probably an upper estimate of the real deactivation rate of the current.

### Alanine Induced Current in Homo-Exchange Mode

**Figure 8:**
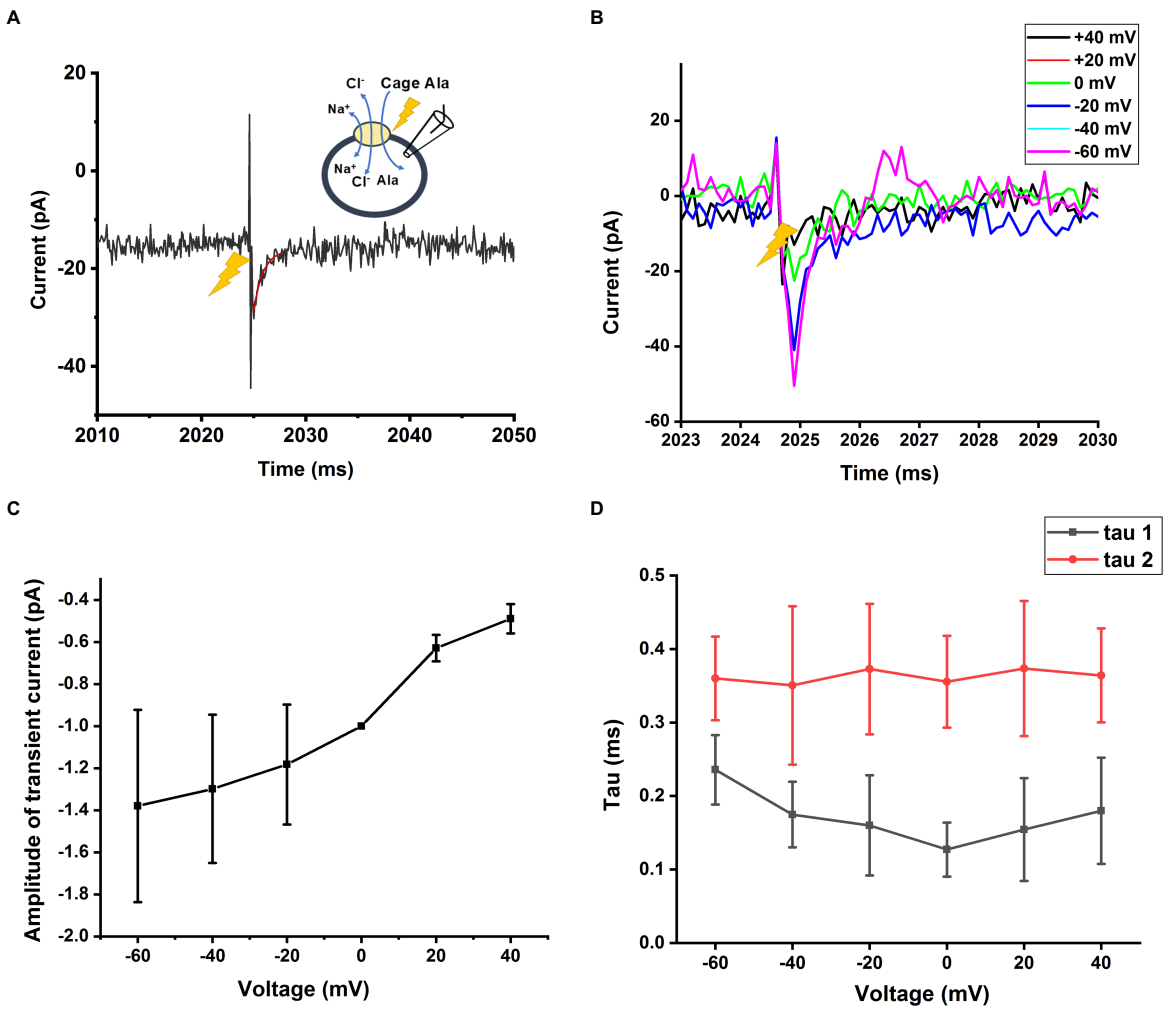
Transient current is also present in homo-exchange mode. **(A)** Transient inward current upon caged alanine photolysis (2mm) applied to the outside of the cell was also observed in the sodium/alanine exchange mode. The time of the laser flash is indicated by the arrow. The external solution contained 140mM NaCl, the internal; solution 130mM NaCl and 10mM alanine. **(B)** Voltage dependence of the transient alanine-induced inward current in exchange mode. The voltage in **(A)** was 0mV. **(C)** The amplitude of the transient current from **(B)** is plotted as a function of the transmembrane potential. **(D)** Time constants of the current rise and decay as a function of the membrane potential.

Finally, we measured SLCA14 function under homo-exchange conditions. Here, the intracellular solution contained 130mM NaCl and 10mM alanine (likely saturating concentrations, although the intracellular affinities are not known). Initially, the extracellular solution only contained NaCl (140mm), but no amino acid substrate. It is expected that the amino acid binding site is facing to the outside under these ionic conditions (the transporter can run in reverse under those conditions, but due to almost saturating concentrations of Na^+^ and Cl^−^, steady-state reverse transport current is expected to be small, due to *trans*-inhibition). Next, an alanine concentration jump is performed on the extracellular side, using photolysis of caged alanine, resulting in alanine binding and re-equilibration of the translocation equilibrium. Interestingly, transient inward current was observed under these conditions (Figure 8A), pointing to electrogenicity in the substrate translocation reaction step(s). As expected, little steady state current was observed (Figure 8A), because the reaction is in equilibrium and no net transmembrane flow of charge occurs. We further tested the voltage dependence of the transient currents induced by caged alanine photolysis with membrane potentials ranging from −100 to +60mV in exchange mode. The magnitude of inward currents generated increased with negative transmembrane potential as shown in Figures 8B,C. However, the time constants for the current rise and decay were largely independent of voltage (Figure 8D), indicating that the valence of the partial reaction causing this transient current is low.

## Discussion

Members of the SLC6 transporter family have been well studied, for example, the monoamine transporters, dopamine (DAT), norepinephrine (NET), serotonin (SERT) transporters, and glycine transporters (GlyTs), which are more closely related to SLC6A14. In previous studies (Sloan and Mager, 1999; Nakanishi et al., 2001; Ugawa et al., 2001; Umapathy et al., 2004; Karunakaran et al., 2008), the substrate selectivity of SLC6A14 has been reported. However, the electrophysiological properties and details of the transport mechanism have not been investigated as well as for other members of the SLC6 family. Here, we employed fast-solution exchange methods and rapid amino acid application through laser-photolysis to gain detailed insight into transporter function, mechanism and kinetics. With respect to amino acid selectivity, we found that amino acids with hydrophobic side chain, such as Trp, Leu, Met, Phe, and Ala generally interact with SLC6A14 with the highest apparent affinities, whereas glycine and positively charged amino acids generate the largest transport current at saturating substrate concentrations. These results on substrate selectivity are in agreement with previous reports (Sloan and Mager, 1999; Ugawa et al., 2001; Umapathy et al., 2004; Karunakaran et al., 2008) and highlight the broad recognition range of amino acid substrates for the SLC6A14 family member (Uchiyama et al., 2008).

Other members of the SLC6 family, including the GlyTs, are chloride dependent (Zafra and Gimenez, 1986; Aragon et al., 1987; Liu et al., 1993; Zafra et al., 1997; Lopez-Corcuera et al., 1998; Roux and Supplisson, 2000; Zhang et al., 2021). Consistently, SLC6A14 was also be found to be chloride dependent (Sloan and Mager, 1999; Nakanishi et al., 2001; Hatanaka et al., 2004; Umapathy et al., 2004; Karunakaran et al., 2008). In agreement with these previous results, our data suggest that the nature of the anion in the extracellular solution influences transport function. Here, the presence of external Cl^−^ largely increases transport currents. However, in contrast to previous reports, the amino acid substrate-induced current was not fully abolished in the absence of Cl^−^ (replacement by Gluconate^−^ and Mes^−^). Also, iodide was able to partially substitute for Cl^−^. These results could be interpreted in several ways. First, it is possible that Gluconate- and Mes^−^ could substitute for chloride; however, their significantly larger size and inability to replace Cl^−^ in other transporters makes this possibility less likely. Second, the transporter may have some residual transport activity in the absence of bound Cl^−^. Third, the residual current in the absence of Cl^−^ could be caused by an uncoupled conductance. We favor this last possibility, because in the closely-related glycine transporters, residual current in the absence of Cl^−^ was also observed, but glycine uptake strictly required Cl^−^ (Erdem et al., 2019).

Amino acid transport was previously reported to be Na^+^ dependent, with a stoichiometry of two co-transported Na^+^ ions for each transported amino acid molecule (Nakanishi et al., 2001; Umapathy et al., 2004; Karunakaran et al., 2011). However, SLC6A14 shares the highest sequence identity with GlyT2 (SLC6A5, 53.38% identity), which was reported to have a stoichiometry of 3:1 for Na^+^:substrate cotransport (Roux and Supplisson, 2000). Furthermore, the third Na^+^ binding site residues appear to be conserved between the two transporters, raising the possibility of 3:1 stoichiometry for SLC6A14 also. This idea was confirmed in a recent preprint (Le Guellec et al., 2021). Our data confirm the Na^+^ dependence of SLC6A14, with a Hill coefficient of 1.96, consistent with a Na^+^ coupling stoichiometry of 2, or possibly 3. Experimental Hill coefficients >1, in general, point to a coupling stoichiometry larger than 1, but are a poor measure of the exact coupling ratio.

SLC6A14 also displayed Na^+^ dependent transient currents in response to voltage jumps, similar to other SLC6 family members and amino acid transporters from other families (Mager et al., 1996; Mim et al., 2007; Zhang et al., 2007; Wang et al., 2018, 2020, 2021; Erdem et al., 2019; Zielewicz et al., 2019), including the GABA transporters (Bicho and Grewer, 2005) and GlyT2 (Erdem et al., 2019; Grewer et al., 2000b. These transient currents were present in the absence of substrate, indicating that they are related to Na^+^ binding to the empty transporter, or conformational changes associated with it, for example, a potential redistribution of the inward-outward-facing equilibrium of the empty transporter. The decay of the currents was biphasic, indicating the potential existence of two processes underlying these transient currents, potentially the binding of two Na^+^ ions. The valence of the charge movement was 0.9, suggesting that the underlying process accounts for a substantial amount of the valence of the whole transport cycle. In contrast to the GABA transporter, in which the Na^+^−dependent transient currents decayed on a 100ms time scale, the SLC6A14 transient charge movement was much faster, with a decay time constant of 7.1±1.0ms at 0mV (Figure 6A). This time constant is in line with GlyT2 results, showing 5ms value (Erdem et al., 2019). In contrast, no Na^+^ -dependent transient currents were observed in GlyT1, again highlighting the seemingly close relationship between SLC6A14/GlyT2 sequence and function, with less similarities with GlyT1. As with other transporters, the Na^+^-dependent charge movement allows an estimation of the number of transporters in the cell membrane. With a typical charge movement of 0.98 pC, and a valence of 0.9, the number of active transporters in the cell membrane can be calculated as 6.7×10^6^. Together with a typical transport current of 150 pA, the turnover rate is in the range of 140s^−1^, depending on the number of charges transferred in one transport cycle (see section “Discussion” on Na^+^ stoichiometry above). Turnover rate was also estimated from the decay of current after amino acid removal, a method that was previously applied to GlyTs (Erdem et al., 2019). From these data, the lower limit of the turnover rate is obtained, because of potential limitations by the rate of solution exchange. The turnover rate estimated from this method was >53s^−1^, close to the number from the transient current recordings. These results suggest that SLC6A14 is, to our knowledge, the transporter in the SLC6 family with the highest turnover rate, with other members ranging between 1 and 20s^−1^ (Kristensen et al., 2011).

The results discussed in the previous paragraphs, while important for the details of SLC6A14 kinetics and function, are as expected from previous kinetic analysis of other SLC6 family members. On the other hand, information from pre-steady-state analysis of transport currents is less available, in particular for SLC6A14. Our results from rapid substrate application to the transporter indicate the existence of a rapidly-decaying pre-steady-state current signal before the steady state is reached. This transient current is extremely fast and was not observed with rapid solution exchange, even using piezo-based solution switching. With a time constant of 0.4ms, the rate constant of the underlying transport reaction step(s) is 2,500s^−1^, much faster than the Na^+^-dependent transient current and the turnover rate of the transporter. Therefore, the underlying reaction is not rate limiting for the overall amino acid transport rate. What are the transporter reaction step(s) responsible for this pre-steady-state current signal? Since the transient current is also present in exchange mode, in which the Na^+^ and Cl^−^ concentrations on both sides of the membrane are high, and at least saturating for external ions (the internal binding site affinities are unknown), it stands to reason that states, in which the co-transported ions are dissociated cannot be visited. Therefore, the transient current most likely reflects on partial reactions in the translocation branch of the amino acid transporter cycle, which are likely very fast reaction steps. In addition, these steps contribute to the electrogenicity of the transporter, being responsible for at least a small part of the overall charge movement.

**Figure 9:**
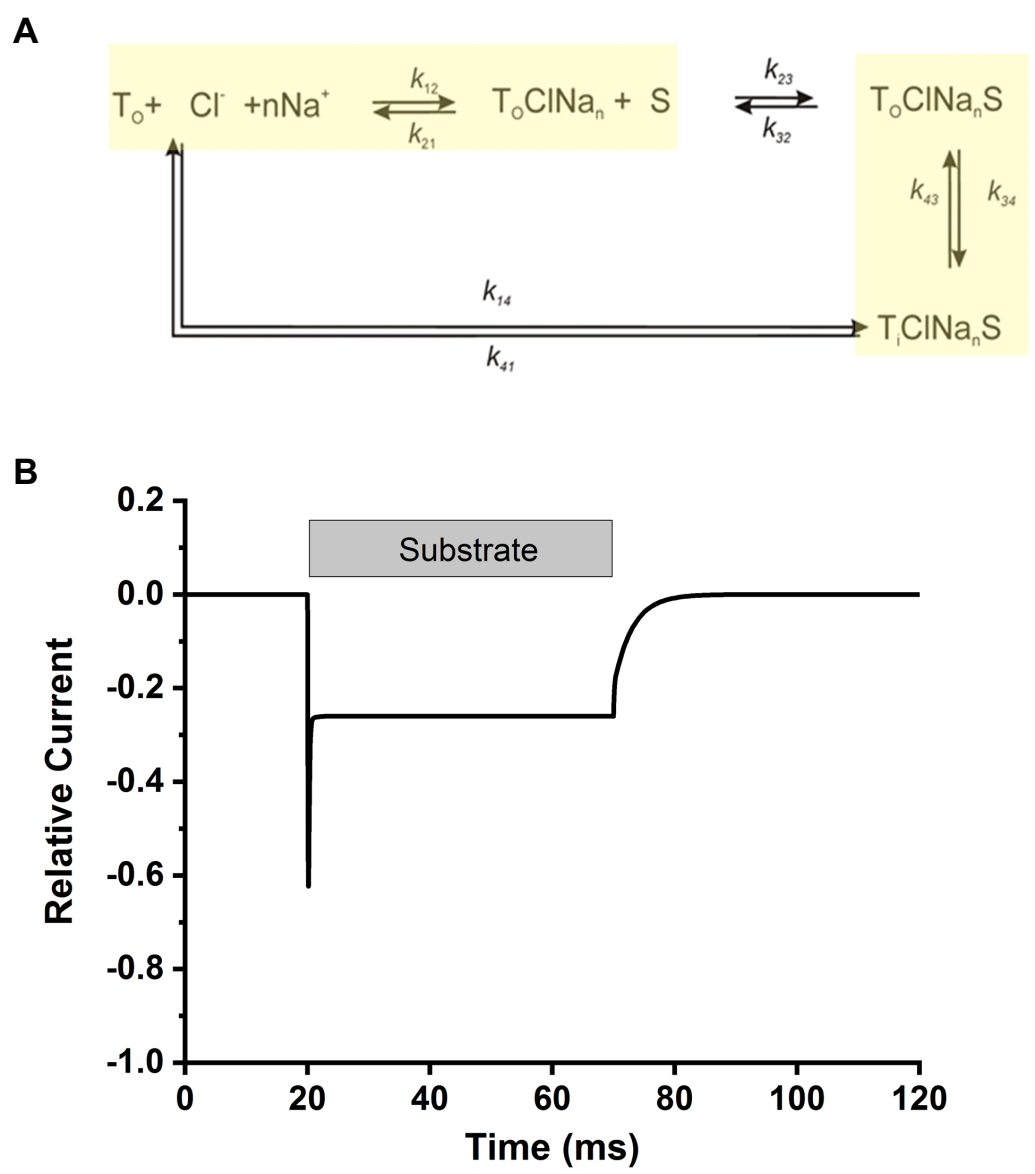
Simplified transport cycle can explain the alanine-induced transient current. **(A)** Simplified kinetic model for substrate transport by SLC6A14. The sodium binding and substrate translocation steps, which were modeled to be voltage dependent, are highlighted in yellow. **(B)** Simulated transport current in response to rapid amino acid substrate application and removal, indicated by the grey bar. Simulations were performed with Berkeley Madonna software, using numerical integration of the differential rate equations pertaining to the kinetic scheme in **(A)**. Ionic conditions were 140mM NaCl and 2mM substrate (external). The kinetic parameters were: k_12_=2M^-1^ms^-1^, k_21_=0.05ms^-1^ (zQ=0.8), k_23_=10,000M^-1^ms^-1^, k_32_=2ms^-1^, k_34_=4ms^-1^, k_43_=4ms^-1^ (zQ=0.4), k_41_=3ms^-1^, k_14_ was set to 0ms^-1^ for forward transport conditions. All simulations were performed at 0mV. Voltage-dependent rate constants was calculated using the equations: ki(V)=ki(0) exp(-ziFV/(2RT)), where i denotes the respective reaction step. F, R and T have their usual meaning. The time-dependent transport current, I(t), was calculated using the following equation: $I\left(t\right)=T_{0}e\left[\Sigma -z_{i}\left(k_{i}P_{i}-k_{i}P_{i+1}\right)\right]$ , where P denotes the fractional population of the respective state, i, as defined in **(A)**. T0 is the number of transporters under observation, e the elementary charge and k_i_ and k_−i_ are the rate constants for binding of the respective transition of valence z_i_.

Overall, our data point to a kinetic model in analogy to other members of the SLC6 family, in particular the closely related glycine transporters (Erdem et al., 2019). Here, Na^+^ and Cl^−^ bind from the extracellular side to the transporter before the amino acid substrate, with Na^+^ binding being a voltage dependent step, contributing majorly to the electrogenicity of the transporter and being a relatively slow step in the transport cycle (Figure 9A). After binding of the ionic co-substrates, the amino acid substrate binds and results in a transporter competent for translocation, a step that is fast and also slightly electrogenic. In this aspect, the mechanism of SLC6A14 appears to be somewhere in-between glycine transporter subtypes 1 and 2, with the former displaying the major electrogenicity in the substrate translocation step(s), whereas the latter shows voltage dependent Na^+^ binding. This simple model can reproduce the major kinetic aspects of transporter behavior upon alanine concentrations jumps, as shown in Figure 9B, with electrogenic steps attributed to Na^+^ binding (valence of 0.8) and, to a lesser extent, to substrate/sodium translocation (valence of 0.4).

Our results highlight the importance of using rapid kinetic techniques, together with kinetic modeling, to investigate partial reactions of secondary-active transporter cycles. While rapid solution exchange methods have been extensively used in the past, their time resolution is too slow to resolve rapid transporter reactions that occur in the low millisecond to sub-millisecond time scale. An excellent example is the SLC6A14 transient, alanine-induced current in forward transport and exchange mode. While the laser photolysis method with caged alanine allows one to temporally resolve the kinetics of this current component, even currents recorded using the very fast piezo-based solution exchange method do not show the transient current. Therefore, based on rapid solution exchange, the false conclusion could be reached that this rapid current component does not exist.

The disadvantage of the laser photolysis method is that caged compounds have to be synthesized first, if they are not commercially available. Generally, neurotransmitters and other biologically important compounds, such as ATP (Mccray et al., 1980), are available in caged form, but this is not the case for amino acids that are substrates for neutral amino acid transporters, such as SLC6A14. Here, we utilized the α-carboxy-nitrobenzyl (CNB) group as a photolabile protecting group for alanine. This group was previously shown to efficiently release amino acids when protected in the α-carboxy position (Grewer, 1999; Grewer et al., 2000a). CNB-caged amino acids are also water soluble and show other desirable properties, such as rapid release of the carboxylate upon photolysis. For example, the analogous CNB-caged glycine displayed release kinetics with time constants in the 5–100μs range, and quantum yields of 0.38 (Grewer et al., 2001). While we did not test the detailed photophysical parameters of CNB-caged alanine, other than the reaction spectrum, we expect these properties to be very similar to CNB-caged glycine. Overall, these results highlight the ease of synthesis of CNB-caged amino acids and their applicability to study neutral amino acid transporter kinetics.

In conclusion, we have characterized the steady-state and pre-steady state kinetics of the neutral/basic amino acid transporter SLC6A14 in detail, providing novel information on the rates of important partial reactions in the transport cycle, and their voltage dependence. In particular, the turnover rate was found to be faster than that of other, previously characterized members of the SLC6 family, including the closely related glycine transporters. In addition, substrate translocation appears to be extremely fast, with a time constant in the sub-millisecond range. Na^+^ binding to the empty transporter is electrogenic, and slower than other partial reaction, potentially contributing to the rate limitation of the transport cycle. In analogy to GlyT1, substrate translocation was also found to be electrogenic. Together, these mechanistic insights contribute to our understanding of SLC6A14 function, and how it plays a part in amino acid homeostasis.

## Data Availability Statement

The raw data supporting the conclusions of this article will be made available by the authors, without undue reservation.

## Author Contributions

YS acquired experimental data, performed data analysis and interpretation, performed kinetic simulations, synthesized caged alanine, and co-wrote the manuscript. JW acquired experimental data, performed data analysis and interpretation, performed kinetic simulations, and co-wrote the manuscript. EN synthesized compounds and planned synthetic strategies. CG conceptualized and directed the project, analyzed and interpreted data, acquired funding, and co-wrote the manuscript. All authors contributed to the article and approved the submitted version.

## Funding

This study was supported by a grant from the National Institutes of Health (R01GM108911) to Avner Schlessinger and CG, and the R15 GM135843-01 awarded to CG.

## Conflict of Interest

The authors declare that the research was conducted in the absence of any commercial or financial relationships that could be construed as a potential conflict of interest.

## Publisher’s Note

All claims expressed in this article are solely those of the authors and do not necessarily represent those of their affiliated organizations, or those of the publisher, the editors and the reviewers. Any product that may be evaluated in this article, or claim that may be made by its manufacturer, is not guaranteed or endorsed by the publisher.
