# Supplementary material for: Pre-steady-state Kinetic Analysis of Amino Acid Transporter SLC6A14 Reveals Rapid Turnover Rate and Substrate Translocation
Source: Front Physiol. 2021 Nov 16;12:777050. doi: 10.3389/fphys.2021.777050 (PMC8637194; doi:10.3389/fphys.2021.777050)
Supplement: Supplementary file 1 [file Data_Sheet_1.pdf]

Supplementary Material

Table S1: Summary of apparent affinities of different amino acids.

Substrates	Experimental K_m (μM)	Substrates	Experimental K_m (μM)
Serine	320 ± 60	Glutamine	6900 ± 3700
Alanine	210 ± 40	Glycine	560 ± 63
Leucine	53 ± 16	Lysine	420 ± 44
Methionine	49 ± 12	Tryptophan	19 ± 3
Valine	140 ± 39	Histidine	160 ± 31
Phenylalanine	43 ± 7	Cysteine	240 ± 44
Arginine	340 ± 67	Asparagine	1200 ± 300

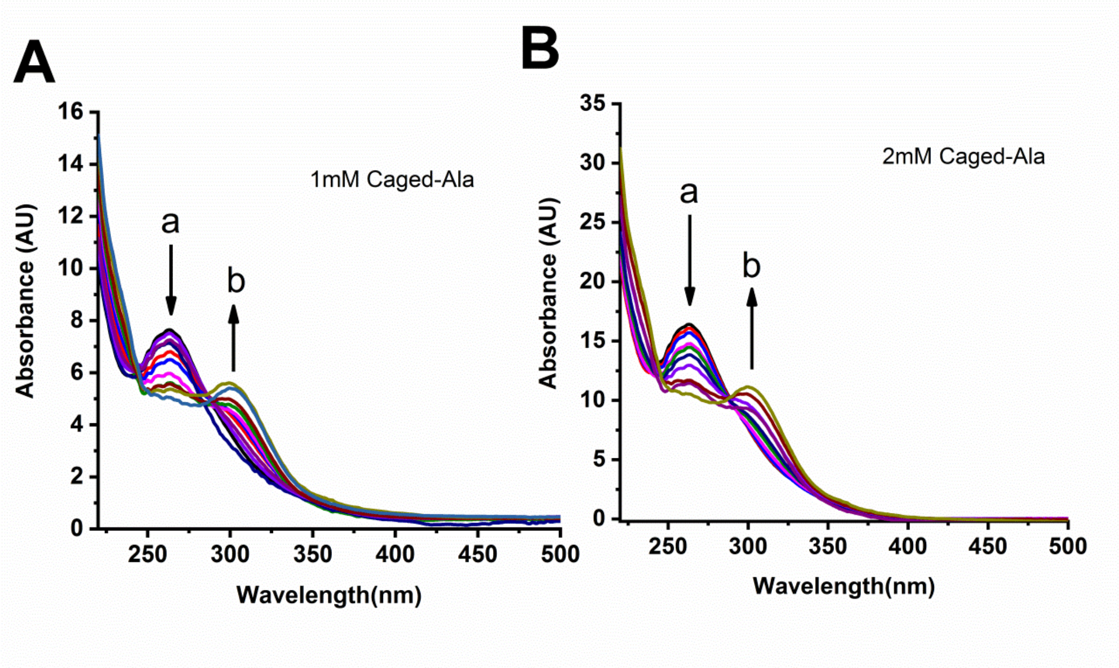

Figure S1 (A, B) 1 mM and 2 mM of cage alanine were subjected to laser irradiation. The absorbance was after intervals of five exposures to the laser flash. The absorbance was measured using BioSpec-nano Shimadzu Spectrophotometer. The peak **a** represents starting material and peak **b** is the photolyzed product.
